# Nitrogen deficiency in barley (*Hordeum vulgare*) seedlings induces molecular and metabolic adjustments that trigger aphid resistance

**DOI:** 10.1093/jxb/erv276

**Published:** 2015-06-02

**Authors:** Gloria Comadira, Brwa Rasool, Barbara Karpinska, Jenny Morris, Susan R. Verrall, Peter E. Hedley, Christine H. Foyer, Robert D. Hancock

**Affiliations:** ^1^Centre for Plant Sciences, Faculty of Biology, University of Leeds, Leeds LS2 9JT, UK; ^2^The James Hutton Institute, Invergowrie, Dundee DD2 5DA, UK

**Keywords:** Cross-tolerance, kinase cascades, metabolite profiles, *Myzus persicae*, nitrogen limitation, oxidative stress, sugar signalling.

## Abstract

Agricultural nitrous oxide (N_2_O) pollution resulting from the use of synthetic fertilizers represents a significant contribution to anthropogenic greenhouse gas emissions, providing a rationale for reduced use of nitrogen (N) fertilizers. Nitrogen limitation results in extensive systems rebalancing that remodels metabolism and defence processes. To analyse the regulation underpinning these responses, barley (*Horedeum vulgare*) seedlings were grown for 7 d under N-deficient conditions until net photosynthesis was 50% lower than in N-replete controls. Although shoot growth was decreased there was no evidence for the induction of oxidative stress despite lower total concentrations of N-containing antioxidants. Nitrogen-deficient barley leaves were rich in amino acids, sugars and tricarboxylic acid cycle intermediates. In contrast to N-replete leaves one-day-old nymphs of the green peach aphid (*Myzus persicae*) failed to reach adulthood when transferred to N-deficient barley leaves. Transcripts encoding cell, sugar and nutrient signalling, protein degradation and secondary metabolism were over-represented in N-deficient leaves while those associated with hormone metabolism were similar under both nutrient regimes with the exception of mRNAs encoding proteins involved in auxin metabolism and responses. Significant similarities were observed between the N-limited barley leaf transcriptome and that of aphid-infested *Arabidopsis* leaves. These findings not only highlight significant similarities between biotic and abiotic stress signalling cascades but also identify potential targets for increasing aphid resistance with implications for the development of sustainable agriculture.

## Highlight

Nitrogen deficiency induces extensive metabolic adjustment in barley leaves mediated largely by sugar signalling and receptor-like kinase cascades that overlap with biotic stress pathways to induce aphid resistance.

## Introduction

Agricultural production is estimated to contribute approximately 10–12% of total anthropogenic greenhouse gas emissions (Burney *et al.*, 2010). The most significant contribution to agricultural emissions arises from the production and use of nitrogen (N) fertilizers. For example the energy intensive Haber-Bosch process for N fixation accounts for more than 50% of the total energy use in commercial agriculture (Woods *et al.*, 2010). Furthermore, N fertilization of agricultural soils results in sharply increased emissions of nitrous oxide (N_2_O). This greenhouse gas has almost 300 times the warming potential of CO_2_ (Shcherbak *et al.*, 2014) and represents almost 40% of direct agricultural greenhouse gas emissions (Burney *et al.*, 2010). There is therefore a clear rationale for the reduction of N use within agricultural production.

Despite these problems, N fertilization continues to be a key factor in crop yield with much of the increase achieved over the last 50 years ascribed to high levels of inorganic N fertilization (Miller and Cramer, 2005). Plants assimilate N from nitrate (NO_3_^-^) or ammonia (NH_4_^+^) that is taken up from the soil. However these chemical species can also be converted to N_2_O by microbial metabolism in a process leading to exponential increases in emissions as N is supplied beyond that required for crop needs (Shcherbak *et al.*, 2014). While plants have highly efficient systems for the uptake of both nitrate and ammonia over a broad range of concentrations (Tsay *et al.*, 2007; Miller *et al.*, 2009) the implications of more precisely controlled N application is that crops may experience relatively short periods of N limitation that induce metabolic readjustments that may impact on crop yields prior to the induction of stress responses and leaf senescence.

The metabolic pathways of primary nitrate assimilation and N cycling in plants are well established. Recent years have seen a large increase in our understanding of the complex regulation of N metabolism and the interactions between N assimilation and other pathways, particularly respiration, photosynthesis and photorespiration (Lewis *et al.*, 2000; Foyer and Noctor, 2002; Foyer *et al.*, 2011). Moreover, the nitrate signalling pathways that modify plant growth and development are becoming increasingly well characterized particularly in terms of the mechanisms that regulate gene expression (Forde, 2002; Vidal and Gutierrez, 2008; Forde and Walch-Liu, 2009; Krouk et al., 2010a, b).

Nitrate-deficient plants develop larger root systems with more extensive branching. In turn shoot growth is restricted under N deficiency and photosynthesis declines because leaf senescence is accelerated (Forde, 2002; Foyer *et al.*, 2003; Scheible *et al.*, 2004). Leaf carbohydrates, particularly starch, accumulate as a result of N deficiency, as do secondary metabolites, such as phenylpropanoids, anthocyanins and flavonoids (Lou and Baldwin, 2004; Fritz *et al.*, 2006).

Transcriptome profiling has been extensively used over the last 15 years to identify N-responsive genes (Wang et al., 2000, 2003, 2004; Scheible *et al.*, 2004; Hermans *et al.*, 2006; Krouk *et al.*, 2010a). The combination of transcriptome and metabolite data has also increased our understanding of the links between gene expression and metabolism (Hirai *et al.*, 2004; Peng *et al.*, 2007; Simons *et al.*, 2014). Such studies have shown that nitrate deficiency stimulates the expression of genes involved in amino acid catabolism, protein degradation, autophagy and ubiquitin-proteasome pathways as well as genes encoding phenylpropanoid metabolism, while genes encoding photosynthetic proteins and biosynthetic pathways that require N-containing metabolites tend to be down-regulated (Peng *et al.* 2007; Schlüter *et al.*, 2012; Amiour *et al.* 2012). While in general, growth is limited by N deficiency with the channelling of available N into essential metabolic processes and defence compounds, plants show a range of adaptive responses. For example, leaf protein contents and high amino acid levels can be maintained under N deficiency if growth rates are slow (Tschoep *et al.*, 2009).

Although the paradigm of N-limited growth, derived from decades of N stress research, is one of retarded growth and development, the real world situation is more complex. Within the context of precision farming, crop species might be expected to undergo periods of N limitation as soil concentrations dwindle followed by periods of N-repletion or N excess. Within this context, advantage might be achieved by maintaining a broader range of metabolic C:N composition. At the early stages of N limitation this mechanism becomes systemic and less selective because of the need for the cells to triage constituents, degrading various components to provide nutrients to permit essential growth and prioritize completion of the life cycle. The following experiments were therefore undertaken in order to characterize the transcript and metabolite profiles of barley leaves in seedlings that define the early responses of leaves to N deficiency, at a point where net photosynthesis was about 50% lower than in N-replete conditions. Barley was chosen for these studies because many of the transcriptome and metabolite profiling studies in the literature characterizing plant responses to N limitation have been performed on model plant species such as *Arabidopsis* (Wang et al., 2000, 2003, 2004; Hirai *et al.*, 2004). Relatively few ‘omic’ studies have been performed on crop species (Jukanti *et al.*, 2008; Amiour *et al.*, 2012; Schlüter *et al.*, 2012, 2013), but the availability of omic techniques in barley allows us to characterize adaptive mechanisms in a non-targeted global analysis.

Given that the metabolic interplay between carbon (C) and N sets the stage for plant productivity and C partitioning and allocation, the following analysis provides new evidence of how anabolic and catabolic processes are co-ordinately managed in barley leaves suffering N limitation. Previous systems analyses of monocotyledonous crop species such as maize to N deficiency have been undertaken following extended periods of N deficiency (Amiour *et al.*, 2012; Schlüter *et al.*, 2012; 2013). The experiments described here were conducted in barley seedlings that were germinated for 7 d in the absence of added N, to a point where the plants had two leaves followed by growth for a further period under different N regimes in the absence or presence of aphids. The data presented demonstrate that the extensive systems reprogramming that occurs during the acclimation of barley leaves to N-limitation also entrains enhanced resistance to phloem-feeding insects. The results presented thus identify putative new molecular markers for improved N use efficiency and stress tolerance traits in crop species.

## Materials and methods

### Plant material and growth conditions

Seeds of barley (*Hordeum vulgare* L. cv. Golden Promise) were germinated for 7 d in the absence of added N. The seedlings were sown in pots in vermiculite in controlled environment chambers with a 16h light/8h dark photoperiod (irradiance 450 µmol m^-2^ s^-1^), 21ºC/16ºC day/night temperature regime and 60% relative humidity. The pots were arranged in trays with 16 pots per tray. Every 2 d each tray was provided with 2 l of a nutrient solution consisting of 0.2mM KH_2_PO_4_, 0.2mM K_2_SO_4_, 0.3mM MgSO_4_·7 H_2_O, 0.1mM NaCl, 0.1 µM MnCl_2_, 0.8 µM Na_2_MoO_4_·2H_2_O, 0.7 µM ZnCl_2_, 0.8 µM CuSO_4_·5H_2_O, 2 µM H_2_BO_3_, 50 µM Fe(III)-ethylenediaminetetraacetic acid (EDTA)-Na and either 5mM KNO_3_ (N replete) or 0.1mM KNO_3_ (N deficient). Plants were grown for 7 d after the initiation of the low and optimal N treatment regimes, to a point where net photosynthesis in N-deficient seedlings was ~50% that of N-replete leaves. Plants were harvested at this point and the following measurements and analyses were performed.

### Shoot and root biomass

Fourteen-day-old seedlings were harvested and separated into shoots and roots. These were weighed immediately and then dried in an oven at 80ºC for 2 d after which the tissues were weighed again.

### Photosynthesis measurements

Photosynthetic gas exchange measurements were performed using a portable Ciras-2 Infrared Gas Analyser (model ADC 225 Mark 3, The Analytical Development Company Ltd, Hoddesdon, UK) set at 450 μmol m^-2^ s^-1^ photosynthetically active radiation (PAR), 40–50% relative humidity in the leaf chamber, with leaf chamber CO_2_ and O_2_ concentrations maintained respectively at 400±10 μmol mol^-1^ and 210 mmol mol^-1^. The temperature of the leaf chambers was set at 20±0.5ºC. Calculations of net CO_2_ assimilation rate and stomatal closure were performed as described previously (Von Caemmere and Farquhar, 1981).

### Pigment and total protein content

Leaf pigments were extracted and quantified according to the method of Lichtenthaler (1987). Leaf protein was quantified according to the method of Bradford (1976).

### Carbon and nitrogen content

The C and N contents were determined on the dried leaf and root material from five biological replicates per treatment, using a LECO Trumac combustion analyser (Yara UK Limited Company, York, UK).

### Ascorbate, glutathione and pyridine nucleotide assays

Ascorbate, glutathione and pyridine nucleotides were extracted and quantified as described by Queval and Noctor (2007).

### Metabolite analysis by gas chromatography/mass spectrometry (GC/MS)

GC/MS analysis was performed on extracts from four biological replicates per treatment. At the end of the N treatment period, leaves of four individual barley plants were lyophilized and 100±5mg of dried material were weighed into glass tubes. Leaves were sequentially extracted in methanol, water and chloroform for 30min each at 37ºC as previously described (Foito *et al.*, 2013). Internal standards (ribitol and nonadecanoic acid) were added following the initial methanol addition. Finally, an additional aliquot of water was added and the polar and non-polar phases were separated and converted to trimethylsilyl or methyl ester derivatives. Metabolite profiles for the polar and non-polar fractions were acquired following separation of compounds on a DB5-MSTM column (15 m × 0.25mm × 0.25 μm; J&W, Folsom, CA, USA) using a Thermo-Finnigan DSQ II GC/MS system. Data was then processed using Xcalibur software. Peak areas relative to internal standard were calculated following normalization to 100mg extracted material.

### RNA extraction

RNA was extracted from leaves of four individual barley plants treated in parallel to those used for metabolite profiling as described by Kerchev *et al.* (2011).

### Microaarray analysis

Microarray processing was performed on leaf RNA extracts from four biological replicates per treatment, using a custom-designed barley Agilent microarray (A-MEXP-2357; www.ebi.ac.uk/arrayexpress). The microarray contains *~*61 000 60-mer probes derived from predicted barley transcripts and full-length cDNAs (The International Barley Genome Sequencing Consortium, 2012).

These probes were selected from a total of ~80 000 predicted genes by prioritizing them according to their annotation (see section S7.1.4 and figure S18 in supplementary material file of The International Barley Genome Sequencing Consortium, 2012). The high-confidence gene set was used in its entirety (*n*=26 159). This set of predicted genes is based on being supported by homology to at least one closely related species (*Brachypodium distachyon, Sorghum bicolor, Oryza sativa* and *Arabidopsis thaliana*). Next, 14 481 genes were added that had been annotated as ‘remote homologues’, based on a lack of homology to monocot proteins. We also added 7999 genes from the ‘Triticeae-specific’ category, which is defined as having significant BLASTN hits to the wheat fl-cDNA library but no significant BLASTX hits to angiosperm reference protein sequences. The remainder of sequences on the chip (*n*=12 848) derive from genes that had no homology to any of the databases used and are assumed to be specific to barley. This resulted in a total of 61 487 genes represented on the chip by a single probe sequence each.

Microarray processing was performed according to the ‘One-Color Microarray-Based Gene Expression Analysis’ protocol (v. 6.5; Agilent Technologies). Data were extracted using Feature Extraction (FE) software (v. 10.7.3.1; Agilent Technologies) with default settings, and subsequently analysed using GeneSpring GX (v. 7.3; Agilent Technologies) software. Data were normalized using default Agilent FE one-colour settings in GeneSpring and filtered to remove inconsistent probe data flagged as absent in more than one replicate per sample. Probes were identified as significantly changing using one-way Analysis of Variance (ANOVA) with a *P*-value of <0.05 with Bonferroni multiple-testing correction. Raw data can be accessed via the array express website (www.ebi.ac.uk/arrayexpress) using accession number E-MTAB-2242.

For the microarray validation (Supplementary Fig. S1), eight genes were selected based on their different expression and qRT-PCR was performed using the Universal Probe Library (UPL) system (Campbell *et al.*, 2010).

### Aphid bioassays

In order to determine the impact of N status on aphid fecundity, barley plants were germinated and grown in the absence of N for 7 d and then high or low N nutrient solution was applied for a further 7 d as described above. At the end of this period a single one-day nymph of *Myzus persicae* genotype G (Kerchev *et al.*, 2012) reared on *Solanum tuberosum* cv. Desiree was applied to the lamina of the oldest leaf of seven plants and plants were individually caged inside clear plastic containers (10cm internal diameter × 20cm height) capped with a 200 μm mesh. Plants were provided with low- or high-N solution weekly and after 15 d, plants were carefully removed from the cages and aphids present were counted under a hand lens.

## Results

### Leaf chlorophyll and protein were more responsive than net photosynthesis at the early stages of nitrogen limitation

**Fig. 1.**
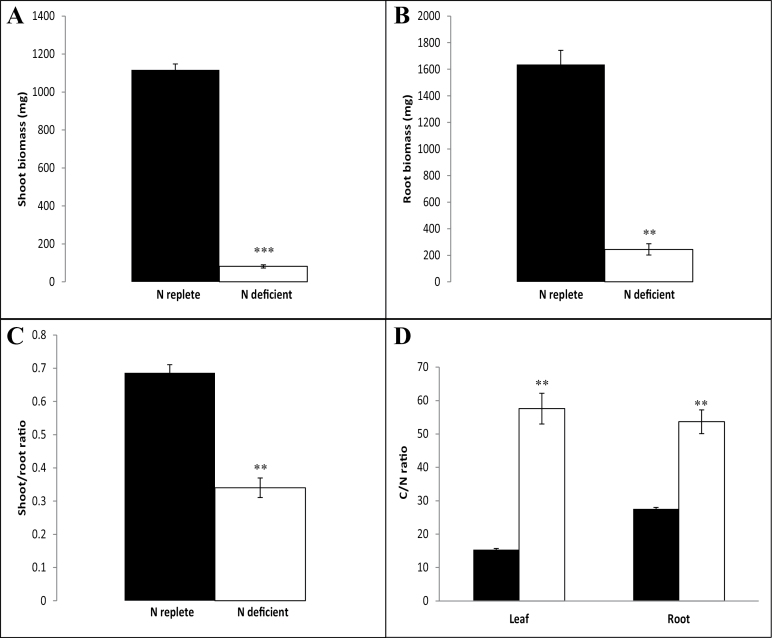
Impact of N availability on plant biomass and C/N ratio. Plants were grown under N repletion or N deficiency as described in the text and harvested 7 d after transfer to variable N conditions. Shoot (A) and root (B) biomass were used to estimate shoot/root ratio (C) and C and N contents were estimated in each as described in the text to calculate C/N ratio (D). Columns represent mean values and bars standard error (*n*=5). In (D) black columns represent plants grown under N repletion and white columns plants grown under N deficiency. Significant differences were estimated using the student’s *t*-test; ** *P*<0.01, *** *P*<0.001.

Seedlings that had been germinated for 7 d in the absence of added N and then grown for 7 d under N deficiency (0.1mM KNO_3_) showed marked differences in leaf chlorophyll and protein contents relative to leaves of plants that had been grown for 7 d under N-replete conditions (5mM KNO_3_). After this period, the seedlings had two leaves under both N regimes. However, the leaves of the plants that had been grown for 7 d under N deficiency were considerably smaller than those grown under N-replete conditions (Supplementary Fig. S2). Shoot (Fig. 1A) and root (Fig. 1B) biomass accumulation were significantly lower in plants that had been grown for 7 d under N deficiency than N-replete controls. Moreover, shoot/root ratios were significantly lower in plants under N deficiency than N-replete controls (Fig. 1C). The total N content of the shoots and roots was significantly lower in plants under N deficiency [0.68±0.09% and 0.78±0.07% (w/w), respectively] than N-replete controls [2.55±0.07% and 1.46±0.01% (w/w), respectively]. These data corroborate results previously observed in maize grown for longer periods under N deficiency (Amiour *et al.*, 2012; Schlüter *et al.*, 2013) and indicate that a reduction in leaf N content occurs at an early stage following transfer to N-deficient conditions. However the C content of shoots and roots was similar under variable N regimes. Shoots grown under N-replete conditions contained 39.03±0.15% C while those grown under N-deficiency contained 38.45±0.25% C while roots contained 40.30±0.40% C under repletion and 41.30±0.10% C under conditions of deficiency. Hence, the C/N ratios were significantly higher in N-deficient plants than N-replete controls (Fig. 1D).

**Fig. 2.**
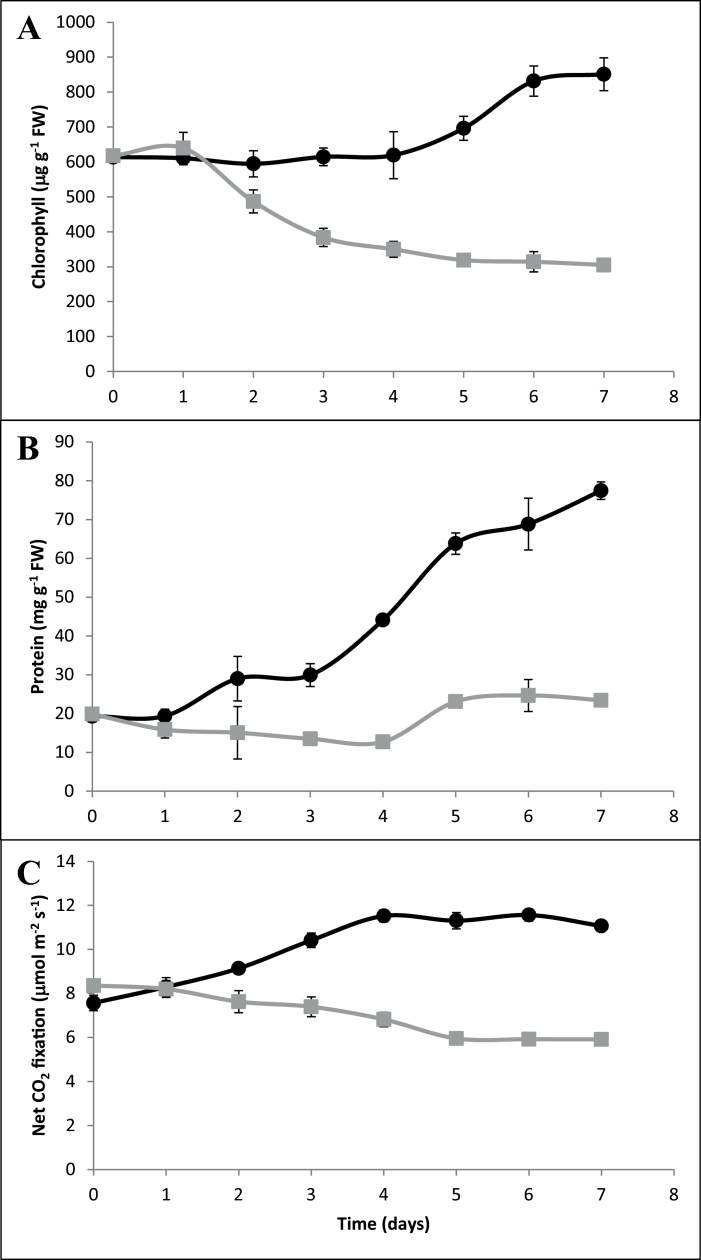
Impact of N availability on leaf chlorophyll, protein and gas exchange in barley. Plants were grown under N repletion or N deficiency as described in the text. Total leaf chlorophyll content (A), protein (B) and net CO_2_ fixation (C) were estimated as described. Points represent mean values and bars standard error (*n*=5). Data from plants grown under N-replete conditions are indicated by black circles and from those grown under N deficiency, grey squares.

Over the 7 d of growth under N deficiency, leaf chlorophyll contents declined relative to N-replete controls, such that at day 7 the leaves of the N-deficient seedlings had only about 40% of the chlorophyll of the N-replete controls (Fig. 2A). Over the same period, leaf protein contents were relatively constant in the N-deficient seedlings (Fig. 2B). In contrast, leaf protein contents were significantly increased in the leaves of seedlings grown for 7 d under N-replete conditions (Fig. 2B). Thus, at day 7 leaf protein was ~75% higher in the N-replete controls than in leaves of plants grown under N deficiency (Fig. 2B).

Net photosynthesis rates were high in the leaves of seedlings that had been germinated for 7 d in the absence of added N (Fig. 2C). Thereafter, net photosynthesis rates slowly declined in the leaves of plants grown for 7 d under N deficiency. In contrast, net photosynthesis rates gradually increased in the N-replete controls over the same time period (Fig. 2C). Hence, by day 7 under the different N regimes photosynthetic CO_2_ assimilation rates were ~50% lower in the leaves of N-deficient seedlings than N-replete controls (Fig. 2C). We chose the 7-d time-point for the transcriptome and metabolite profiling analysis because it would capture the relatively early responses to N limitation rather than the later responses, which would be largely dominated by features associated with senescence and cell death.

### Leaf redox balance was maintained at the early stages of nitrogen limitation

**Fig. 3.**
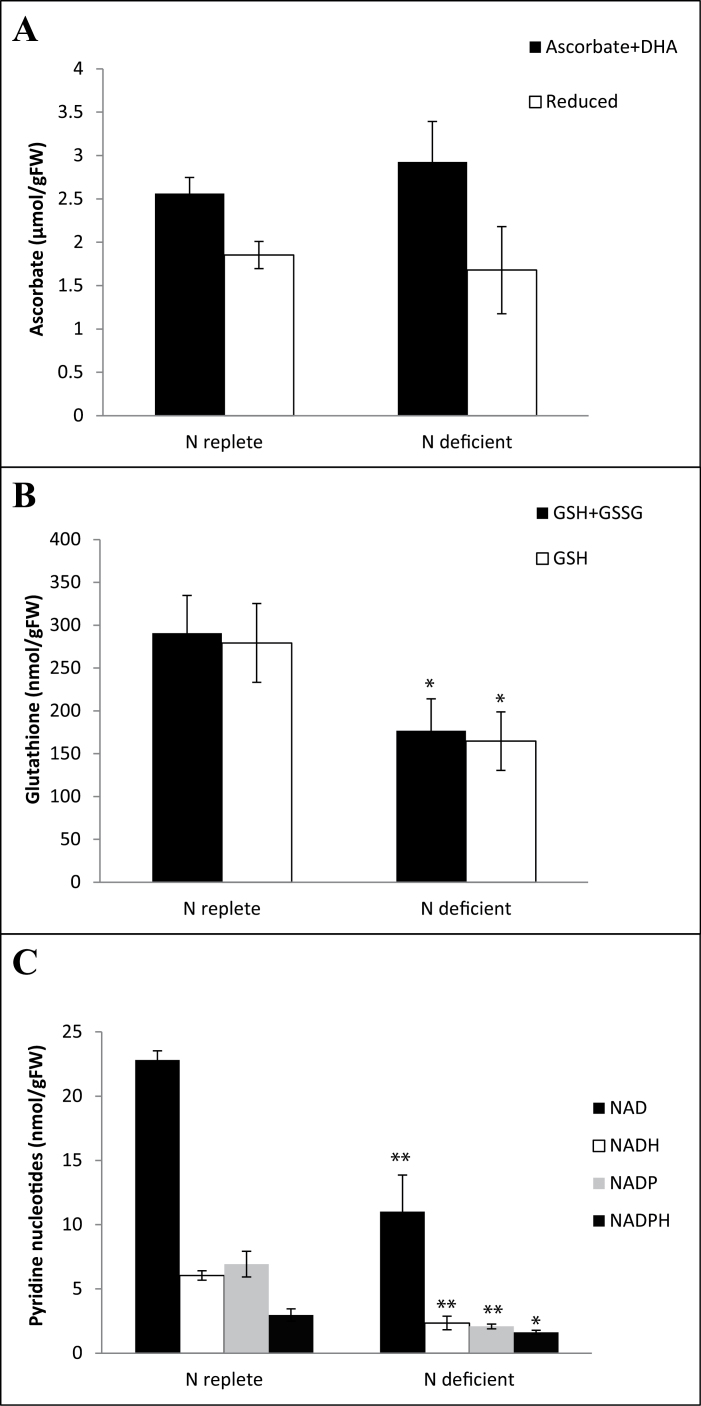
Impact of N availability on shoot redox couples. Plants were grown under N repletion or N deficiency as described in the text and harvested 7 d after transfer to variable N conditions. Total and reduced ascorbate (A), glutathione (B) and oxidized and reduced pyridine nucleotides (C) were estimated according to Queval and Noctor (2007). Data is represented according to the figure legends as mean values ±SE (*n*=4) and significant differences between plants grown under N repletion or N deficiency as estimated by the Students *t*-test are indicated; * *P*<0.05, ** *P*<0.01.

The leaves had similar amounts of total and reduced ascorbate under both growth conditions (Fig. 3A). In contrast, the levels of N containing redox buffers were significantly lower in leaves grown under N deficiency with reductions observed for total and reduced glutathione (Fig. 3B) as well as for pyridine nucleotides (Fig. 3C). Despite the reduced concentrations of N-containing redox buffers ratios of reduced to oxidized forms were not significantly different. GSSG represented <10% of the total glutathione pool under both conditions (4.71±1.78% and 6.70±0.64%, *P*=0.35 under N-replete or N-deficient conditions, respectively). Similarly, NADH/NAD ratios were not significantly different under different N conditions (0.27±0.02 and 0.24±0.06, *P*=0.68 under N-replete or N-deficient conditions, respectively). Although NADPH/NADP ratios tended to be higher under N deficiency (0.81±0.14) than under N-replete conditions (0.43±0.06) this was not significant according to the Student’s *t*-test (*P*=0.08).

### Global impacts on the leaf transcriptome at the early stages of nitrogen limitation indicate extensive systems rebalancing

**Fig. 4.**
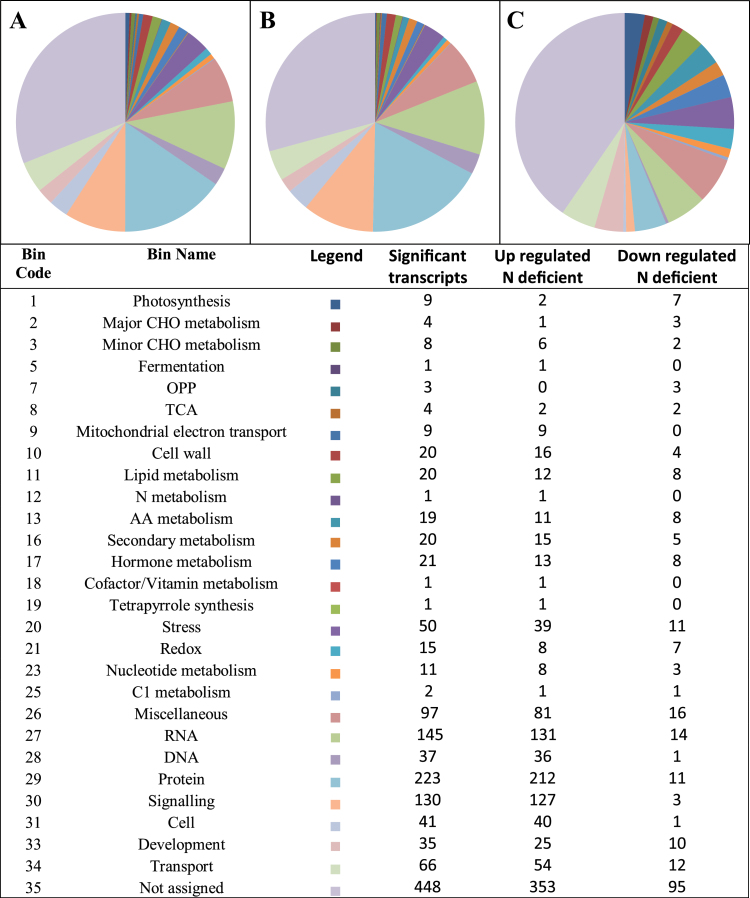
Functional classification of transcripts exhibiting significantly different abundance under N-replete or N-deficient conditions. Venn diagrams illustrate the relative numbers of transcripts that were significantly differentially abundant (A) or those more (B) or less (C) abundant under N deficiency. The table lists the MapMan bins containing differentially abundant transcripts and provides the number of transcripts in each bin.

A transcript profile comparison was performed on leaves after 7 d of growth under N-deficient and N-replete conditions. Analysis of variance indicated that N nutrition had a significant impact on the accumulation of 1443 transcripts (*P*<0.005) of which 1206 were significantly more abundant under N limitation (Supplementary Table S1). Functional analysis of the transcript dataset revealed that of the subset of transcripts that were more abundant under N limitation there was enrichment in transcripts associated with a number of metabolic functions (Fig. 4). Large numbers of transcripts associated with signalling (MapMan bin 30) and protein metabolism (MapMan bin 29) were significantly differentially abundant under conditions of N metabolism as were those associated with RNA metabolism (MapMan bin 27), transport (MapMan bin 34) and stress (MapMan bin 20). Similar results were observed in maize that had been subjected to long-term N stress where transcripts associated with signalling, stress and transport were highly abundant in leaves (Amiour *et al.*, 2012). In order to analyse the metabolic functions that were affected by N deficiency in greater detail, a PageMan analysis was conducted to examine the transcript groups that were significantly over-represented under conditions of N deficiency (Supplementary Fig. S3). This confirmed over-representation of transcripts associated with cell signalling (MapMan bin 30) particularly those associated with a number of receptor-like kinase families (MapMan bin 30.2) amongst the subset of transcripts that were more abundant under N deficiency. Furthermore, this analysis revealed a de-repression of transcripts associated with sugar and nutrient signalling (MapMan bin 30.1) under N-depleted conditions relative to those under N-repletion. Other functional groups strongly represented in the more abundant under N limitation transcript subset included those encoding ATP-dependent metalloproteases (MapMan bin 29.5.9) found exclusively in mitochondria and chloroplasts. Furthermore transcripts associated with secondary metabolism were over-represented in the more abundant under N limitation subset of transcripts, particularly those associated with phenylpropanoid biosynthesis (MapMan bin 16.2).

Amongst the group of transcripts that were significantly over-represented in the pool that were significantly less abundant under N limitation were those associated with photosynthesis (MapMan bin 1), particularly those encoding for components of PSII (MapMan bin 1.1). Similarly, transcripts associated with C mobilization such as those required for sucrose (MapMan bin 2.2.1) and lipid degradation (MapMan bin 11.9) were significantly over-represented in the pool of transcripts that were less abundant under N limitation.

Relative to leaves under N-limiting conditions, a number of metabolic functions were over-represented in the subset of transcripts that were significantly more abundant in N-replete leaves. These included transcripts associated with amino acid biosynthesis, particularly those associated with synthesis of aspartate family amino acids (MapMan bin 13.1.3). Similarly, transcripts associated with the accumulation of storage proteins (MapMan bin 33.1), auxin metabolism and responses (MapMan bin 17.2) and glutaredoxins (MapMan bin 21.4) were over-represented in N-replete leaves. Serine proteases (MapMan bin 29.5.5) were significantly over-represented in the group that were lower in abundance under N limitation.

### Rebalancing thylakoid composition at the early stages of nitrogen limitation

**Fig. 5.**
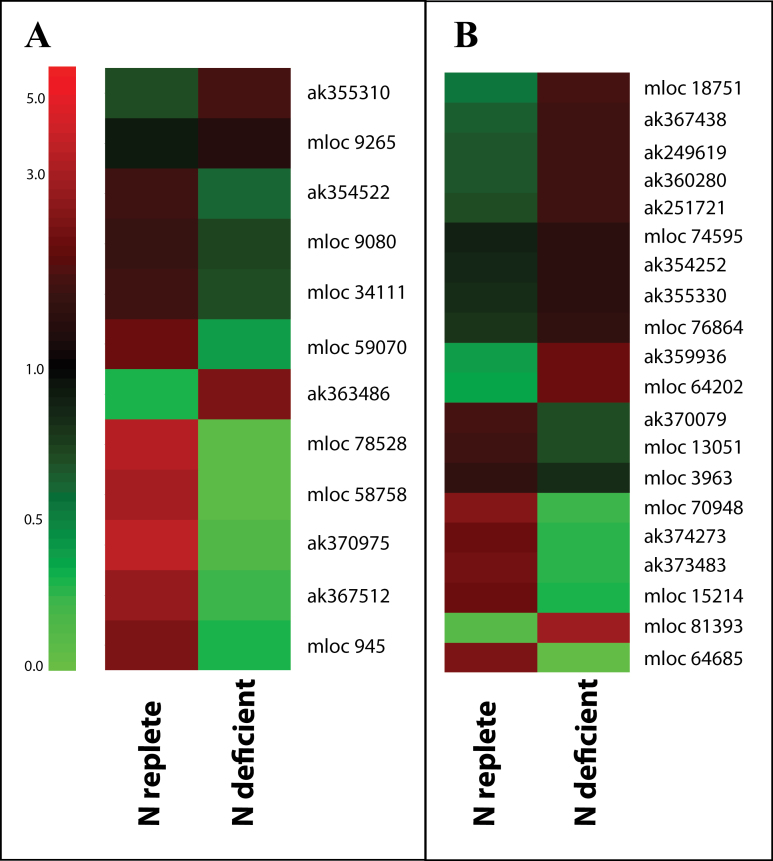
Cluster analysis comparison of abundance of transcripts encoding proteins associated with photosynthesis (A) or lipid metabolism (B) in barley leaves grown under N-replete or N-deficient conditions. Relative transcript abundance is illustrated on a red (high) to green (low) scale. Significantly differentially abundant transcripts associated with photosynthesis (A; MapMan bin 1) or lipid metabolism (B; MapMan bin 11) are presented as heatmaps showing relative transcript abundance according to the green-red scale indicated in A. Accession numbers of individual transcripts are indicated to the right of each figure.

Further analysis of transcripts associated with primary metabolism suggested a down-regulation of transcripts associated with PSII that are consistent with reduced C assimilation resulting from reduced photosynthetic electron transport. For example, transcripts encoding structural components of PSII such as a transcript encoding a PSII 10kDa polypeptide (ak354522) and several chlorophyll a/b binding proteins (mloc 78528, mloc 58758, mloc 945, ak370975) were significantly less abundant in leaves under N limitation (Fig. 5A, Supplementary Table S2). Transcripts encoding components of the photosynthetic electron transport chain such as apocytochrome f (mloc 34111) and proteins required for ATP synthesis (mloc 9080) were also significantly less abundant in leaves under N limitation. In contrast, transcripts encoding proteins associated with cyclic electron flow such as a transcript encoding an NADPH dehydrogenase (mloc 9265) and the regulation of electron transport such as a protein with similarity to the *Arabidopsis* ACCLIMATION OF PHOTOSYNTHESIS TO ENVIRONMENT 1 (ak363486) were significantly more abundant under N limitation. Consistent with a rebalancing of thylakoid composition, transcripts encoding ATP-dependent metalloproteases (AAA proteases) were significantly over-represented amongst transcripts increased in abundance under N-limitation (Supplementary Fig. S3, bin 29.5.9). These mitochondrial and chloroplastic membrane-bound proteases have roles in the biogenesis and degradation of PSII (Chi *et al.*, 2012). Phytol and related metabolites were significantly lower in leaves suffering N limitation relative to N-replete controls (Table 1, Supplementary Table S3). Consistent with reduced CO_2_ fixation and a loss of source function, a number of transcripts encoding invertases showed altered abundance following N limitation (ak355310, ak367512, mloc 59070).

**Table 1.** Influence of N availability on phytol content in barley leaves

**Phytol**	**Relative concentration** ^*a*^		**Ratio N replete: N limited**	**Significance** ^***b***^
	**N limited**	**N replete**		
Phytol A	1.04±0.25	1.81±0.58	1.74	0.29
Phytol B	1.83±0.55	4.19±0.65	2.29	0.04
Phytol C	0.61±0.21	1.72±0.37	2.82	0.05
Phytil methyl ether	85.97±39.47	206.41±43.01	2.40	0.10
Phytil methyl ether 2	86.30±39.73	202.83±41.50	2.35	0.10

^*a*^ Mean phytol content ±SE (n = 4) is reported as peak area relative to the internal standard.

^*b*^ Statistical significance level as estimated using the Student’s *t*-test.

### Systems triage rebalancing primary and intermediary leaf metabolism at the early stages of nitrogen limitation

Transcripts such as mloc 15214 and mloc 3963 that encode proteins associated with phospholipid synthesis were significantly less abundant in leaves under N limitation (Fig. 5B, Supplementary Table S4). In contrast, ak359936 a transcript that encodes a phospholipase was significantly more abundant under these conditions, as was ak354252, which encodes a protein with dual 3-hydroxyacyl-CoA dehydrogenase/enoyl-CoA hydratase activity that is required for the β-oxidation of fatty acids. However, two transcripts encoding proteins required for the synthesis of fatty acids were more abundant under N limitation (ak355330, ak360280), as were a number of transcripts encoding proteins involved in the synthesis of other lipids (mloc 74595, mloc 76864, ak249619, ak367438). However, few changes were observed in the fatty acid content of N-deficient leaves, the only significant difference being observed for tetracosanoic acid (C24:0) that was 1.8-fold more abundant (Supplementary Table S3). Glycerol and glycerol-3-phosphate, essential components of glycerolipids were highly elevated in N-limited leaves (Supplementary Table S3).

**Fig. 6.**
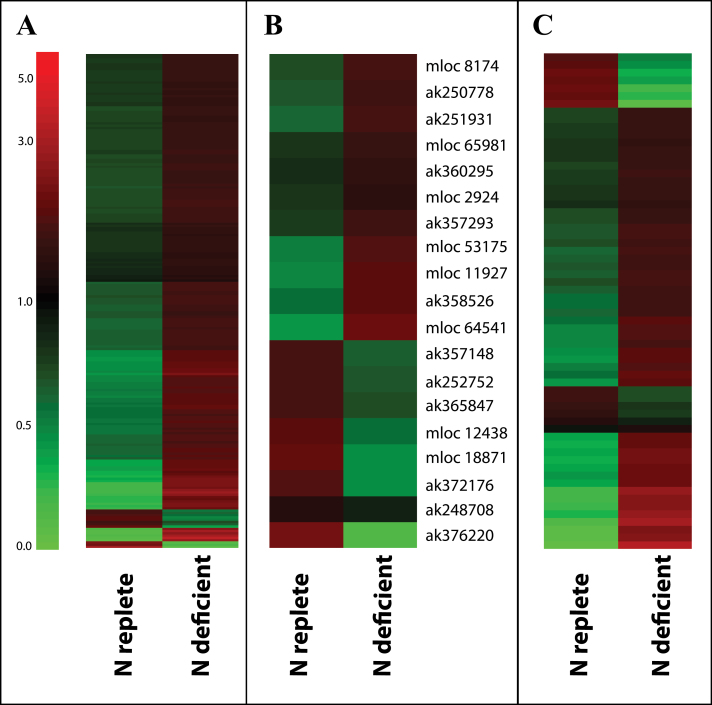
Cluster analysis comparison of abundance of transcripts encoding proteins associated with protein metabolism (A), amino acid metabolism (B) or transport (C) in barley leaves grown under N-replete or N-deficient conditions. Relative transcript abundance is illustrated on a red (high) to green (low) scale. Significantly differentially abundant transcripts associated with protein metabolism (A; MapMan bin 29), amino acid metabolism (B; MapMan bin 13) or transport (C: MapMan bin 34) are presented as heatmaps showing relative transcript abundance according to the green-red scale indicated in A. Accession numbers of individual transcripts associated with amino acid metabolism are indicated to the right of panel B, for transcripts associated with protein metabolism or transport refer to Supplementary Tables 5 and 7 respectively.

Nitrogen limitation increased the abundance of transcripts associated with protein metabolism (Fig. 6A), such as proteases, and large numbers of proteins associated with ubiquitin-mediated proteasome activities (Supplementary Table S5). The abundance of many amino acids such as lysine, tyrosine, methionine, isoleucine, glycine, proline, phenylalanine, asparagine, leucine, threonine and γ-aminobutyric acid (GABA) was greatly increased in leaves under N limitation relative to N-replete controls (Table 2). These data are in marked contrast to previous studies in maize that indicated an almost universal reduction in amino acids (Amiour *et al.*, 2012; Schlüter *et al.*, 2012). The divergence in results may represent differences between a metabolic adjustment and triage phase examined in the present work and longer-term steady state adjustment studied previously. Nitrogen-limitation dependent increases in other amino acids such as glutamate, aspartate and alanine were less marked (Table 2).

**Table 2.** Influence of N availability on amino acid content of barley leaves

Amino acid^a^	**Relative concentration** ^*b*^		**Ratio N limited: N replete**	**Significance** ^***c***^
	**N limited**	**N replete**		
Lys	31.65±9.89	0.32±0.18	99.84	0.087
Tyr	24.16±6.29	0.50±0.25	48.61	0.064
Met	6.47±1.14	0.15±0.06	44.28	0.031
Ile	12.80±2.62	0.42±0.19	30.34	0.041
Pro	11.02±2.92	0.42±0.20	26.24	0.068
GABA	109.22±24.13	8.02±4.50	13.61	0.048
Gly	19.47±2.78	1.84±0.80	10.59	0.001
Val	11.39±1.49	1.18±0.43	9.68	0.015
Phe	22.00±2.21	2.85±0.88	7.71	0.001
Asn	0.55±0.06	0.07±0.03	7.57	0.001
Leu	28.24±9.49	4.48±3.84	6.30	0.049
Thr	4.22±0.66	0.98±0.36	4.33	0.006
Gln	2.72±0.46	0.86±0.23	3.16	0.011
Glu	46.03±25.51	29.94±6.80	1.59	0.488
Oxo-proline	17.21±2.85	10.91±5.28	1.58	0.389
Ala	0.06±0.02	0.05±0.01	1.4	0.492
Asp	17.47±5.27	12.93±4.53	1.35	0.543

^*a*^ Amino acids are indicated using the standard three letter code. GABA, γ-Amino butyric acid.

^*b*^ Mean amino acid content ±SE (*n*=4) is reported as peak area relative to the internal standard.

^*c*^ Statistical significance level as estimated using the Student’s *t*-test.

The levels of a number of TCA cycle intermediates, sugars and their derivatives were significantly higher in leaves suffering N deficiency (Table 3). Similarly, there was a trend towards higher concentrations of compounds metabolically derived from sugars such as inositol, glycerate and threonate under conditions of N deficiency. Relatively few transcripts encoding proteins associated with amino acid activation, protein synthesis and protein targeting were significantly changed in abundance in leaves suffering N deficiency (Supplementary Table S5). Transcripts encoding proteins associated with amino acid metabolism exhibited a less uniform pattern of abundance with some transcripts being increased as a result of N limitation while others were significantly reduced (Fig. 6B, Supplementary Table S6). However, within this pattern of transcript abundance those associated with branched-chain amino acid biosynthesis were mostly more abundant under N limitation. These included ak358526 encoding a putative dihydrodipicolinate reductase, ak360295 encoding a putative acetolactate synthase, ak251931 encoding a putative branched chain amino acid transaminase, mloc 65981 encoding a putative cysteine synthase and mloc 11927 encoding a putative threonine aldolase. This data was consistent with the observed increases in all of the branched-chain amino acids which were between 6- and 30-fold more abundant under conditions of N-limitation (Table 2). On the contrary, mloc18871 and ak376220 encoding an aspartate kinase and a dual function aspartate kinase/homoserine dehydrogenase were significantly less abundant in N-deficient leaves as was ak248708 encoding a putative plastidic aspartate aminotransferase involved in pathways of N assimilation.

**Table 3.** Influence of N availability on content of metabolites associated with sugar and carboxylic acid metabolism in barley leaves

**Metabolite**	**Relative concentration** ^*a*^		**Ratio N limited: N replete**	**Significance** ^*b*^
	**N limited**	**N replete**		
**TCA Intermediates**				
Succinic acid	4.03±0.80	0.16±0.06	25.18	0.039
Malic acid	455±29	30.27±6.67	15.03	0.001
Citric acid	552±164	60.51±12.24	9.13	0.095
Fumaric acid	1.08±0.24	0.43±0.17	2.53	0.071
**Sugars and related metabolites**				
Galactose	64.08±14.99	0.54±0.26	119.11	0.051
Fructose	901±63	27.46±8.52	32.80	0.005
Threonic acid	3.90±1.22	0.17±0.05	22.95	0.092
Inositol	56.24±11.54	2.96±0.41	18.99	0.044
Glucose	1092±78	57.86±14.83	18.87	0.004
Maltose	3.84±0.62	0.22±0.10	17.38	0.025
Glyceric acid	48.72±22.11	4.84±1.61	10.05	0.185
Sucrose	1184±486	450±53	2.63	0.27

^*a*^ Mean metabolite content ±SE (*n*=4) is reported as peak area relative to the internal standard.

^*b*^ Statistical significance level as estimated using the Student’s *t*-test.

Changes in levels of primary metabolites and transcripts associated with primary metabolism were accompanied by an increase in abundance of transcripts associated with transport (Fig. 6C, Supplementary Table S7). In particular, all of the significantly expressed transcripts encoding sugar transporters were significantly more abundant under N limitation. For example mloc 57236 and ak359096 encoding proteins with homology to *Arabidopsis* SUGAR TRANSPORTER 1 were 3–5-fold more abundant under N limitation while ak369803 encoding a protein homologous to *Arabidopsis* SUGAR TRANSPORTER 13 was >10-fold more abundant under the same conditions. Similarly, the majority of amino acid transporters (mloc 16705.3, ak252705, mloc73950.1, mloc 51108.1, ak369769, ak368895) and all but one (mloc 66939.1) of the significantly expressed transcripts encoding peptide transporters were more abundant under N limitation.

Nitrogen limitation had a variable impact on the abundance of transcripts associated with redox processes (Supplementary Fig. S4, Supplementary Table S1). Three transcripts encoding thioredoxins (Trx) were significantly more abundant under N deficiency (mloc 10762 homologous to At3g08710 encoding TrxH9, ak362034 homologous to At2g01270 encoding quiescin-sulfhydryl oxidase 2 and ak372587 homologous to At2g18990 encoding a thioredoxin domain containing protein) while transcripts encoding a cytosolic dehydroascorbate reductase (ak359422) and a mitochondrial/plastidal monodehydroascorbate reductase (ak357867) involved in the regeneration of oxidized ascorbate were significantly less abundant under N deficiency. The impact of N deficiency on the abundance of transcripts encoding glutaredoxins (Grx) was more variable with some transcripts being significantly more abundant (mloc 62185, mloc 13482) and others significantly less abundant (mloc 75646, mloc 55037, mloc 28768).

### Secondary metabolism is enhanced as an early response to nitrogen limitation

Transcripts encoding proteins involved in both the mevalonate (mloc 62766) and non-mevalonate (mloc 63954) pathways of terpenoid synthesis were significantly more abundant in N-deficient barley leaves (Supplementary Table S8). Similarly, the majority of transcripts associated with phenylpropanoid metabolism that were significantly regulated by N availability were more abundant under N deficiency. These included a number of transcripts encoding phenylalanine ammonia lyase (PAL) (ak248841, ak250690, ak250100, ak353809), considered to be the enzyme controlling entry into phenylpropanoid biosynthesis, as well as two transcripts encoding dihydrokaempferol reductase family proteins (ak365198, ak366300). In contrast, two transcripts associated with lignin biosynthesis encoding a protein with strong homology to *Arabidopsis* PAL2 (mloc 6761) and a caffeoyl-CoA O-methyltransferase (mloc59507) were significantly less abundant under N deficiency although a transcript encoding a cinnamyl alcohol dehydrogenase (mloc 43485) was more abundant.

### Signalling cascades underpinning the systems triage that rebalances primary and secondary metabolism in the early responses to nitrogen limitation

**Fig. 7.**
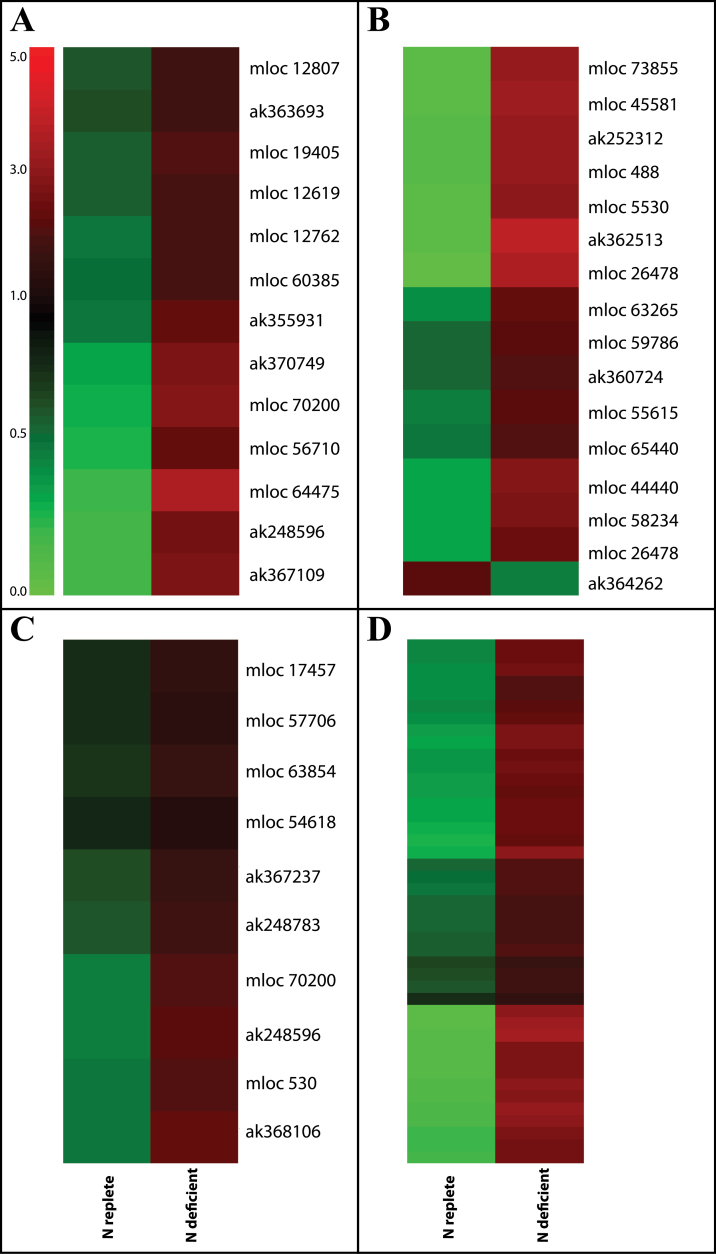
Cluster analysis comparison of abundance of transcripts encoding receptor-like kinases. Relative transcript abundance is illustrated on a red (high) to green (low) scale. Significantly differentially abundant transcripts associated with leucine-rich repeat XI (A; MapMan bin 30.2.11), wall-associated (B; MapMan bin 30.2.25), glycoprotein-like (C: MapMan bin 30.2.24) or DUF 26-containing (D: MapMan bin 30.2.17) kinases are presented as heatmaps showing relative transcript abundance according to the green-red scale indicated in A. Accession numbers of individual transcripts associated with amino acid metabolism are indicated to the right of each figure with the exception of panel D for which transcripts are listed in Supplementary Table 9.

Large numbers of transcripts encoding proteins associated with the post-translational modification of proteins were altered in abundance in leaves suffering N limitation, particularly protein kinases and protein phosphatases. These included transcripts encoding receptor-like kinases with the vast majority of transcripts being more abundant under these conditions. Classes that were highly represented included transcripts encoding leucine-rich repeat XI kinases (Fig. 7A), wall-associated kinases (WAKs) (Fig. 7B), glycoprotein-like kinases (Fig. 7C) and a large number of transcripts encoding kinases containing the domain of unknown function 26 (Fig. 7D) (Supplementary Table S9). Other transcripts associated with signalling whose abundance was dependent on N status included those associated with calcium and G-protein signalling. A number of transcripts associated with hormone signalling, particularly auxin signalling, were also significantly affected by leaf N status as were a small number of transcripts associated with the metabolism of brassinosteroids, cytokinin, ethylene and jasmonate (Supplementary Table S1). Auxin is considered a key player in the regulation of N acquisition and control of morphological responses to N limitation (Kiba *et al.*, 2011)

A large number of transcription factors exhibited differential abundance with respect to N (Supplementary Table S10). Transcripts that were more abundant under N deficiency included RAV2, 1× bHLH, 1× Zn finger, MYB (4, 38 + 3 MYB related), HSF A4A and several WRKYs. In contrast, several transcripts encoding AP2-type and bHLH transcription factors (including PIF4) were lower in barley leaves under N deficiency as were transcripts encoding a JUMONJI (JmjC) domain-containing protein that generally function as histone demethylases.

### Nitrogen deficiency has impacts on biotic interactions

**Fig. 8.**
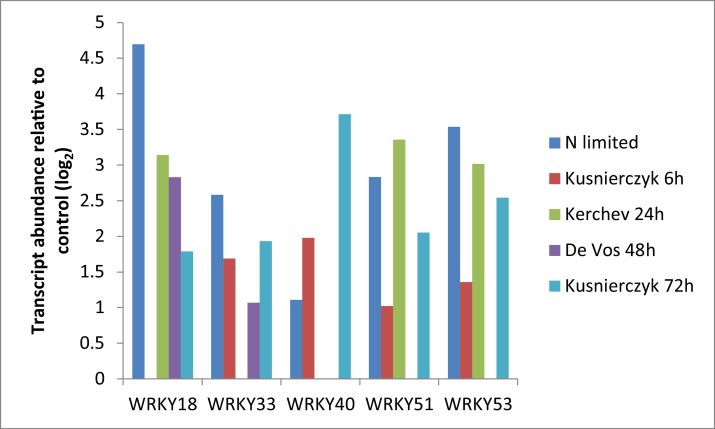
Expression of transcripts encoding WRKY transcription factors following N limitation in barley leaves or aphid infestation of *Arabidopsis* leaves. The expression level of transcripts encoding WRKY18 (mloc70190, At4g31800), WRKY33 (ak376482, At2g38470), WRKY40 (mloc 60890, At1g80840), WRKY51 (mloc 81131, At5g64810) and WRKY53 (ak369804, At4g23810) relative to N-replete or aphid-free controls, respectively, are indicated on a log_2_ scale. Aphid data refers to *Brevicoryne brassicae* infestation of ecotype Landsberg erecta for 6h (Kusnierczyk 6h), *Myzus persicae* infestation of ecotype Columbia for 24 or 48h (Kerchev 24h, De Vos 48h) or *B. brassicae* infestation of ecotype Columbia for 72h (Kusnierczyk 72h). Transcript data following aphid infestation was obtained fromKusnierczyk et al. (2008,^2011^), Kerchev *et al.* (2013) and De Vos *et al.* (2005).

Given the large increases in free amino acids observed in N-deficient barley leaves, we hypothesized that they would represent an excellent nutrient source for the aphid *Myzus persicae* in comparison to leaves grown under N-replete conditions. In order to test this hypothesis, one-day-old nymphs were transferred to N-limited or N-replete leaves and the rate of colony expansion estimated by counting the total number of aphids present after 15 d. Surprisingly, while the number of aphids on N-replete leaves was 16.00±0.78 (*n*=7), no aphids were found on the N-deficient leaves indicating that the progenitor aphids failed to survive until maturity. We observed overlap between gene expression profiles of N-deficient barley leaves and leaves of *A. thaliana* plants infested with aphids (Foyer *et al.*, 2015). In particular, transcripts associated with common signalling pathways were up-regulated under either N-limitation or aphid infestation. The data presented here shows that there is a significant over-representation of transcripts encoding domain of unknown function 26 (DUF26) receptor-like kinases (MapMan bin 30.2.17) as well as those encoding WAKs (MapMan bin 30.2.25) (Supplementary Fig. S3). Similar transcripts were also over-represented following aphid infestation of *Arabidopsis* leaves (Foyer *et al.*, 2015). Downstream signalling networks also shared overlap following N-limitation or aphid infestation, with transcripts encoding WRKY transcription factors significantly over-represented under both treatments (Supplementary Fig. S3; Foyer *et al.* 2015). In particular, transcripts encoding WRKY 18, 33, 40, 51 and 53 were significantly induced following either N-limitation of barley leaves or aphid infestation of *Arabidopsis* leaves (Fig. 8). While *M. persicae* infestation effectively suppressed the expression of transcripts associated with phenylpropanoid and specifically flavonoid metabolism in *Arabidopsis* (Foyer *et al.* 2015), N-limitation had the opposite impact on gene expression in barley where transcripts associated with phenylpropanoid and flavonoid metabolism were over-represented in N-limited leaves (Supplementary Fig. S3, bins 16.2 and 16.8, respectively).

## Discussion

There is general agreement that rates of crop N fertilization can have an impact on aphid fecundity, with significant decreases in fecundity associated with the highest rates of fertilization, as well as with N deficiency (Gash, 2012). However, there are relatively few studies in the literature concerning how plant N fertilization affects susceptibility to aphid infestation, particularly in cereals. The data presented here demonstrate that the relationship between plant N and aphid infestation is complex and not related simply to the N requirement in the insect diet. In particular, coordinated regulation of anabolic and catabolic processes that underpins acclimation to low N-availability confers aphid resistance.

The data demonstrate that photosynthesis and associated pathways were decreased in barley leaves suffering N limitation. However in contrast to studies on long-term N limitation in maize, there is no evidence of oxidative stress and the leaves maintained a large pool of amino acids and TCA cycle intermediates. The N- and metabolite-rich state may be required to support cell division and cell expansion in the developing areas at the leaf base. While the leaves exhibit very low chlorophyll contents, a classic hallmark of leaf senescence (Pintó-Marijuan and Munné-Bosch, 2014), the leaf soluble protein levels remain largely constant during the period of N deficiency, although the values are much lower than those of leaves grown under N-replete conditions.

In photosynthetic N assimilation, the enzymes of the pathway use the reducing power generated by the photosynthetic electron transport chain to reduce nitrate to ammonia and to assimilate ammonia into amino acids. Photosynthesis was significantly lower in the barley leaves suffering N deficiency but there were no indications in our metabolite profiling analysis that this led to increased oxidative stress. Cellular redox homeostasis is maintained by low molecular weight antioxidants such as ascorbic acid and the thiol tripeptide reduced glutathione (GSH), together with antioxidant enzymes and redox-active proteins such as Grxs, Trxs and their associated light- or NADPH-dependent reductases. The antioxidant systems not only prevent oxidative stress but they also participate in signal transduction during biotic and abiotic stresses. Cysteine residues on proteins, such as peroxiredoxins, glutaredoxins, GSH (thiol) peroxidases and Trx, and on GSH mediate oxidative signal transduction leading to the activation of defence responses (Foyer and Noctor, 2009, Mhamdi *et al.*, 2010; Han *et al.*, 2013). The data presented here demonstrate that some transcripts encoding antioxidant proteins such as three Trxs were higher in N-deficient leaves than N-replete controls, as were transcripts encoding proteins associated with stress such as heat shock proteins. These results are indicative of an oxidative signalling response. However, the relative abundances of reduced ascorbate to its oxidized form, DHA, and of GSH relative to the oxidized form, glutathione disulfide (GSSG), were similar in leaves under N-replete conditions and under N deficiency. The absence of significant oxidation of the ascorbate and glutathione pools suggests that cellular redox state was not greatly perturbed by N deficiency. Moreover, while total levels of glutathione and pyridine nucleotides were significantly reduced, the ratios of reduced to oxidized forms were unchanged in the leaves suffering N deficiency, suggesting that reducing power remains sufficient to drive metabolism under these conditions. There is no doubt that primary N assimilation is driven by reducing power in higher plants and GS and GOGAT have been reported to be targets of Grx (Rouhier *et al.*, 2005) and/or Trx (Balmer *et al.*, 2003; Buchanan and Balmer, 2005). The findings of the present study suggest that it is unlikely that oxidative inactivation of N assimilation occurs in barley leaves experiencing N deficiency.

The lower photosynthetic CO_2_ assimilation rates in N-deficient leaves together with the lower levels of chlorophyll and metabolites such as the phytols, are consistent with the observed changes in transcripts encoding components of the photosynthetic electron transport system. However, some transcripts encoding photosynthetic proteins such as the small subunit of ribulose-1,5-bisphosphate carboxylase/oxygenase, components of the PSII oxygen evolving complex and the chloroplast NADH complex were more abundant under N deficiency, suggesting a regulated rearrangement of the photosynthetic system rather than a simple dismantling of the photosynthetic processes at the early stages of low N-induced leaf senescence. The observed changes in transcripts associated with lipid metabolism are consistent with this conclusion. We presume that an increased breakdown of membrane lipids and associated β-oxidation of fatty acids may be required for the generation of reducing power and high energy phosphate groups if the capacity of the photosynthetic electron chain is restricted by N limitation. The data presented here also suggest the presence of more subtle changes in lipid metabolism. Few significant changes were observed in fatty acid profiles between N-replete and N-deficient leaves (Supplementary Table S3). However transcripts associated with lipid metabolism did exhibit significant differences in abundance under the different conditions of N availability (Supplementary Table S4). This included a number of transcripts encoding proteins involved in the synthesis of what might be termed ‘exotic’ lipids (mloc 74595, mloc 76864, ak249619, ak367438). It is therefore possible that the synthesis of these exotic lipids is required to stabilize membranes following the removal of phospholipids. Large increases in levels of free glycerol and glycerol-3-phosphate (G3P) were observed under N limitation, consistent with high rates of membrane lipid turnover. Similarly, free glucosyl/galactosyl-glycerol as well as galactose was highly elevated under N limitation, consistent with the degradation of thylakoid membranes that contain up to 80% galactosylglycerolioids (Hölzl and Dörmann, 2007).

A large number of transcripts associated with protein metabolism, a further hallmark of the early stages leaf senescence, increased in abundance as a result of N limitation. The significant impact of N limitation on protein turnover is witnessed by the increased abundance of transcripts encoding various classes of proteases, together with large numbers of proteins associated with ubiquitin-mediated degradation. These increases are consistent with the observation that free amino acids were significantly higher under conditions of N limitation relative to N sufficiency (Table 2) and support the hypothesis that the failure of leaves to accumulate protein under conditions of N limitation (Fig. 2B) is a specifically evolved strategy rather than simply a result of unavailability of the amino acid building blocks. An enhanced activation of protein degradation systems (in the absence of changes in the levels of transcripts encoding enzymes of N assimilation and amino acid metabolism) may account for the observed differences in amino acid pools in the leaves of N-deficient and N-replete seedlings. The data presented here, collected at an early stage of N limitation, is in marked contrast to that obtained following extended N stress in maize where not only amino acids but also many sugars and TCA-cycle intermediates were significantly reduced in N-limited compared with N-replete leaves (Amiour *et al.*, 2012; Schlüter *et al.*, 2012, 2013). This highlights the significance of not only the plant developmental stage in defining the response to N stress as previously observed (Amiour *et al.*, 2012) but also indicates the segmentation of the plant response to the duration of N stress. The changes in amino acids are indicative of tissues that are rich in available N, a situation that may be required to support the growth of the leaf base. There is a large increase in GABA in the N-deficient leaves, in line with previous observations regarding low N-induced leaf senescence in *A. thaliana* (Diaz *et al.*, 2005; Watanabe et al., 2013) and maize (Schlüter *et al.*, 2012). The functions of GABA in plants remain poorly understood, although it is generally accepted that the GABA shunt can be used to provide succinate in times of stress. Increasing evidence suggests that GABA might also be an important signal molecule with roles in the regulation of C/N interactions in response to stress (Bouché and Fromm, 2004).

The large numbers of transcripts encoding protein kinases and phosphatases that were altered in abundance following the imposition of N-limitation suggest that protein phosphorylation and dephosphorylation plays a significant role in the redirection of metabolism following imposition of this stress. Interestingly, two transcripts homologous to SUCROSE NON-FERMENTING1-RELATED PROTEIN KINASES (SNRKs) (mloc 63787 homologous to SNRK2.5 and mloc 52019 homologous to SNRK3.2) were significantly reduced in abundance following the imposition of N stress (Supplementary Table S1). Taken together, these data suggest that sugar-related protein kinase-mediated cascades and related pathways of signal transduction are altered as a result of N deficiency. Relatively few transcripts encoding components of hormonal synthesis and signalling were altered in abundance in barley leaves as a result of N deficiency, however transcripts associated with auxin metabolism and signalling were well represented. This is consistent with previous data indicating a relationship between the auxin concentration in roots and phloem sap and plant N status (Kiba *et al.*, 2011). Transcripts encoding components involved in other hormone-signalling pathways, particularly salicylic acid and abscisic acid are notable by their absence.

A key question that has always concerned both agronomists and plant scientists, is whether plants suffering abiotic stress become more susceptible to insects and other pests. Aphids feed exclusively on phloem and as such their diet is highly imbalanced with the high sugar content representing a significant osmotic barrier, which is exacerbated by the low phloem N content requiring the ingestion of large volumes to allow the insects to acquire sufficient N (Douglas, 2006). Within this context, it is possible that the amino-acid rich leaves of N-limited plants will provide a better host than N-replete plants. Extensive transcriptome data is available concerning the response of plants to infestation by *M. persicae* (Foyer *et al.*, 2015). Furthermore, although not the most suitable host for *M. persicae*, barley will support colonies of this aphid (Davis and Radcliffe, 2008). We therefore chose to examine the impact of N status on the capacity of barley plants to support *M. persicae* colonization. Contrary to our expectations, while aphids reared on N-replete plants were able to successfully complete their life cycles, one-day-old nymphs transferred to N-deficient plants failed to reach maturity.

It has previously been demonstrated that the specialist aphid *Rhopalosiphum padi* performed more poorly on N-limited barley seedlings, associated with an increased time to locate the phloem and a decreased amino acid content of the phloem (Ponder *et al.*, 2000). However, in their study the resistance was much less significant, with aphid population growth rates being about 10% lower in N-deficient barley seedlings compared with those provided with sufficient N. Furthermore, in these experiments seedlings were grown under N-limiting conditions for much longer periods than the experiments described here and contrary to the data presented here, tissues were amino-acid poor. These data and the fact that the previous experiments were undertaken using a specialist aphid suggest that mechanisms of resistance are likely to diverge. A second potential nutritional influence on resistance in our experiments is the observed increase in leaf sucrose concentration, which may represent an increased osmotic barrier to phloem feeding. However, while leaf sucrose content increased ~2.5-fold (Table 3) in N-limited leaves, the content of phloem mobile amino acids (Winter *et al.*, 1992) increased up to 100-fold (Table 2). These data indicate that sufficient N could be acquired from reduced phloem ingestion and suggest that the osmotic barrier may therefore not be as high as indicated from sucrose measurements alone.

The transcriptome analysis undertaken here raises the possibility that aphid resistance in N-deficient barley plants may be associated with the activation of similar signalling cascades following N restriction or aphid infestation. A comparison of the aphid-induced transcriptome (Foyer *et al.*, 2015) revealed overlap with that of the low N transcriptome (Supplementary Table S1) with WAKs, kinases containing domain of unknown function 26 (DUF26 kinases) and several WRKY transcription factors highly induced by both stresses. These data add support to the previously stated hypothesis that WAKs, DUF26 kinases and WRKY transcription factors play important roles for basal resistance to aphids (Foyer *et al.*, 2015).

A notable difference between the aphid induced transcriptome and that induced by N-limitation was the observation that *M. persicae* feeding suppressed transcripts associated with phenylpropanoid metabolism (Foyer *et al.* 2015) while low N induced such transcripts (Supplementary Fig. S3, bin 16.8). Several previous studies have implicated polyphenols and their oxidation products in aphid resistance (Miles and Oertli, 1993; Lattanzio *et al.*, 2000; Kerchev *et al.*, 2012) and the induction of transcripts associated with polyphenol metabolism under low N conditions may be a contributing factor to aphid mortality.

In addition, the present analysis of the low N metabolome indicates a significant increase in the levels of free G3P. This metabolite was ~250 times more abundant in N-limited than in N-replete leaves. G3P has been demonstrated to act downstream of azelaic acid in systemic acquired resistance (SAR) (Yu *et al.*, 2013). Local synthesis of G3P is required to stimulate systemic resistance to biotrophic bacterial and fungal pathogens (Chanda et al., 2008, 2011). Furthermore, resistance can be induced following external supply of glycerol that is subsequently metabolized to G3P (Chanda *et al.*, 2008). Our results therefore demonstrate that low N-induced increases in G3P accumulation are associated with resistance to aphids. This finding suggests that G3P signalling initiates broad-spectrum resistance against biotrophs and provides a metabolic link between biotic and abiotic signalling pathways.

The data presented here demonstrate that there is a general systemic remobilization of essential C and N resources in leaves experiencing N limitation because of the need to triage constituents to permit essential growth and prioritize completion of the life cycle. In this situation, the regulation of the plant metabolism not only becomes focused on the single goal of completing a reproductive cycle to disperse progeny that can germinate when conditions have improved, but also provides protection of the vegetative tissues against attack by insects such as aphids. The metabolite profiles demonstrate that cell functions acclimate to low N availability by limiting photosynthesis but not respiration, primary N assimilation, or protein turnover. The metabolome changes observed in these studies are consistent with those reported in *A. thaliana* (Diaz *et al.*, 2005; Watanabe et al., 2013) and comparison with other studies in monocotyledonous species (Amiour *et al.*, 2012; Schlüter *et al.*, 2012, 2013) suggest a progressive shift in metabolism dependent on the period of N stress. Early responses to low N in barley leaves result in a metabolite-rich state in which amino acids and sugars are abundant. The previously unforeseen consequence of the extensive metabolic reprogramming triggered by low N nutrition is the accumulation of putative signalling molecules required for the induction of systemic immunity. This intriguing observation opens potential new avenues of investigation, particularly with regard to the possible relationships between G3P signalling and aphid resistance.

## Supplementary data

Supplementary data is available at JXB online.

Supplementary Figure S1. Comparison of transcript abundance as estimated by qRT-PCR and microarray analysis.

Supplementary Figure S2. Appearance of barley plants following germination and growth under N-replete or N-deficient conditions.

Supplementary Figure S3. PageMan representation of gene expression data for barley leaves harvested under N-replete or N-deficient conditions.

Supplementary Figure S4. Cluster analysis comparison of abundance of transcripts encoding redox associated proteins.

Supplementary Table S1. Complete list of transcripts significantly altered in abundance in response to N supply.

Supplementary Table S2. Transcripts associated with photosynthesis or major carbohydrate metabolism significantly altered in abundance in response to N supply.

Supplementary Table S3. Relative concentration of a range of polar and non-polar compounds in barley leaves under N limitation and N repletion.

Supplementary Table S4. Transcripts associated with lipid metabolism significantly altered in abundance in response to N supply.

Supplementary Table S5. Transcripts associated with protein metabolism significantly altered in abundance in response to N supply.

Supplementary Table S6. Transcripts associated with amino acid metabolism significantly altered in abundance in response to N supply.

Supplementary Table S7. Transcripts associated with transport significantly altered in abundance in response to N supply.

Supplementary Table S8. Transcripts associated with secondary metabolism significantly altered in abundance in response to N supply.

Supplementary Table S9. Transcripts associated with signalling significantly altered in abundance in response to N supply.

Supplementary Table S10. Transcripts encoding transcription factors significantly altered in abundance in response to N supply.

Supplementary Data
