# Supplementary material for: Nitrogen deficiency in barley (Hordeum vulgare) seedlings induces molecular and metabolic adjustments that trigger aphid resistance
Source: J Exp Bot. 2015 Jun 2;66(12):3639–55. doi: 10.1093/jxb/erv276 (PMC4463806; doi:10.1093/jxb/erv276)
Supplement: Supplementary Data [file supp_66_12_3639__index.html]

Nitrogen deficiency in barley (Hordeum vulgare) seedlings induces molecular and metabolic adjustments that trigger aphid resistance — Nitrogen deficiency in barley (Hordeum vulgare) seedlings induces molecular and metabolic adjustments that trigger aphid resistance — Supplementary Data 

# Nitrogen deficiency in barley (*Hordeum vulgare*) seedlings induces molecular and metabolic adjustments that trigger aphid resistance

## Supplementary Data

Data files

- Supplementary Data - Supplementary Data
- Supplementary Data - Supplementary Data
