# Supplementary material for: Nitrogen deficiency in barley (Hordeum vulgare) seedlings induces molecular and metabolic adjustments that trigger aphid resistance
Source: J Exp Bot. 2015 Jun 2;66(12):3639–55. doi: 10.1093/jxb/erv276 (PMC4463806; doi:10.1093/jxb/erv276)
Supplement: Supplementary Data [file supp_erv276_jexbot148049_file001.pdf]

*Journal of Experimental Botany*

Nitrogen deficiency in barley (Hordeum vulgare) seedlings causes molecular and biochemical responses that trigger aphid resistance

Gloria Comadira, Brwa Rasool, Barbara Karpinska, Jenny Morris, Susan R. Verrall, Peter E. Hedley, Christine H. Foyer, Robert D. Hancock

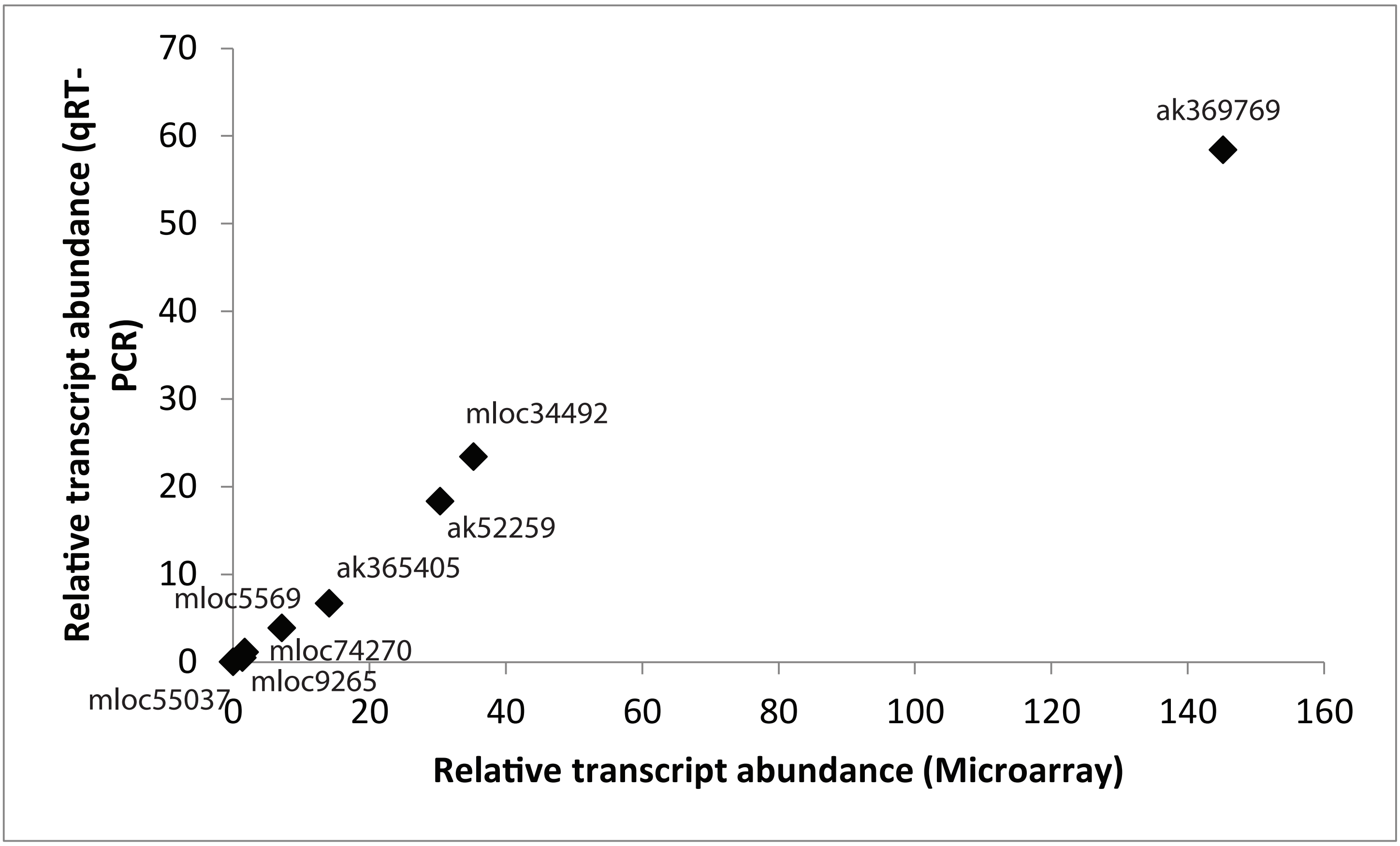


**Supplementary Figure S1.** Comparison of transcript abundance as estimated by qRT-PCR and microarray analysis. Relative transcript abundance of a representative set of transcripts as estimated using microarray analysis or qRT-PCR is shown. The identity of individual transcripts is provided. The correlation ($r^2$) between transcript abundance as estimated by each method was determined to be 0.9691.

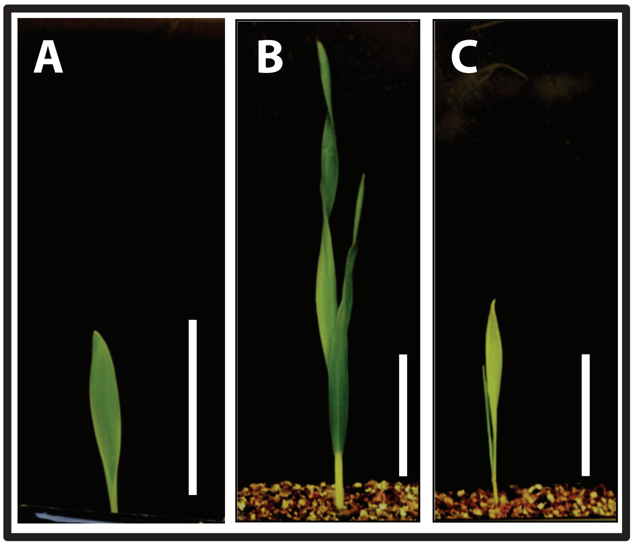


**Supplementary Figure S2.** Appearance of barley plants following germination and growth under nitrogen replete or nitrogen deficient conditions. Barley seeds were germinated and grown on vermiculite in nitrogen free media for 7 days as described (A) then transferred to nitrogen replete (B) or nitrogen deficient (C) media for a further 7 days. Images represent typical plant appearance and scale bars are equivalent to 5 cm.

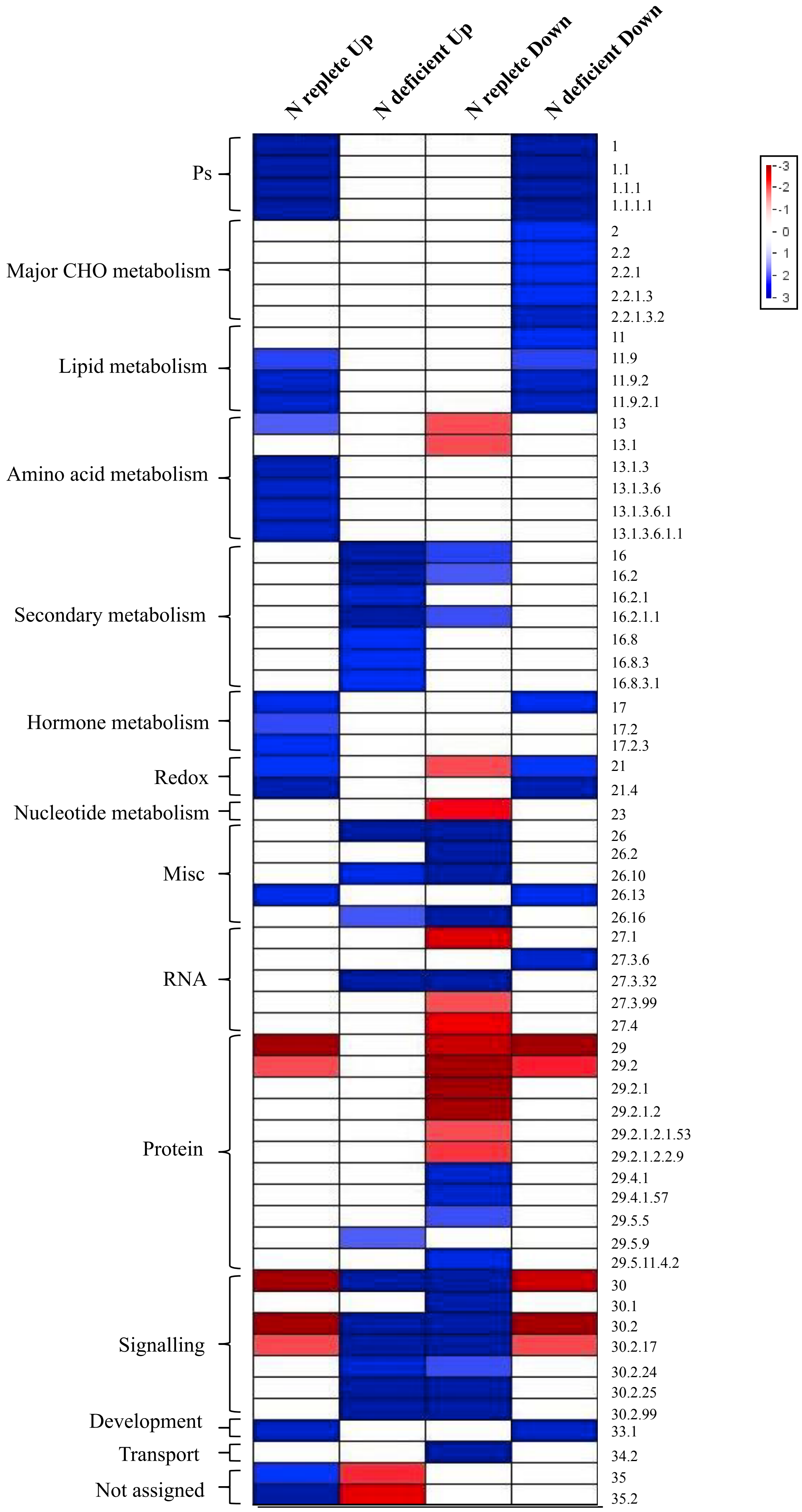


**Supplementary Figure S3.** PageMan representation of gene expression data for barley leaves harvested under N replete or N deficient conditions. Values presented are log $_2$-fold changes for transcripts significantly more (up) or less (down) abundant under the appropriate condition. Bins colored in red are significantly under-represented relative to the rest of the array, whereas Bins colored in blue are over-represented. Major Bins are listed to the left of the figure and individual Bin codes are listed to the right.

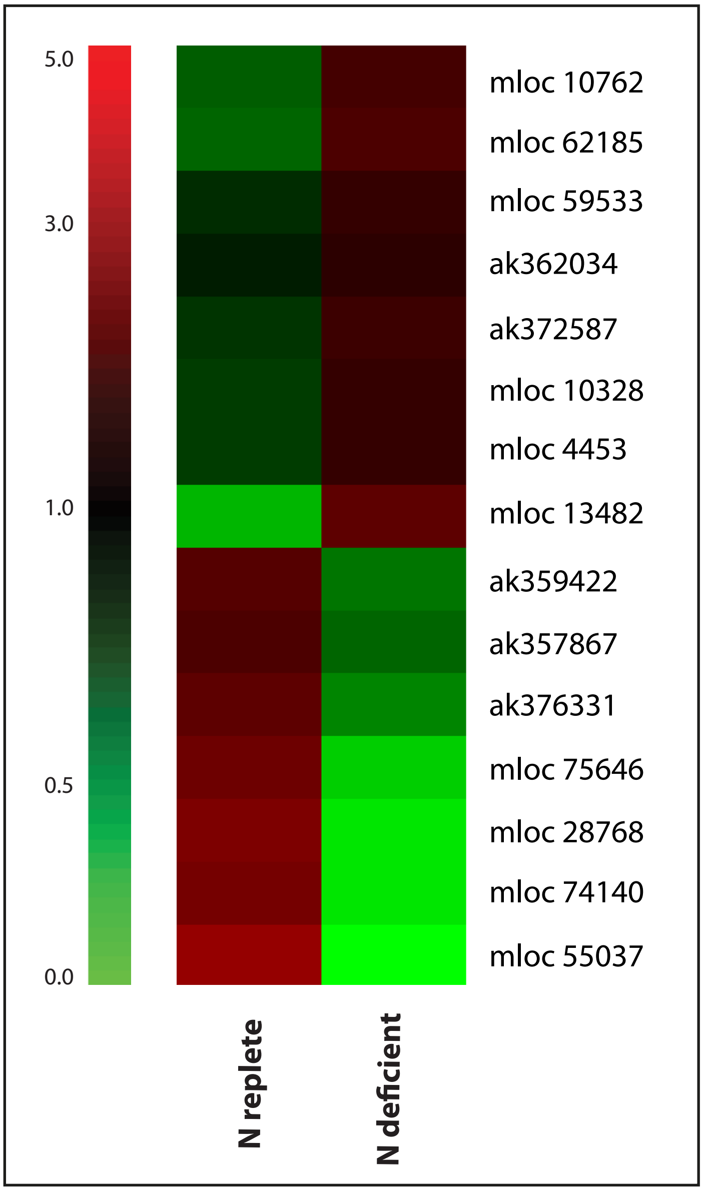


**Supplementary Figure S4.** Cluster analysis comparison of abundance of transcripts encoding redox assoc iated proteins. Relative transcript abundance is illustrated on a red (high) to green (low) scale. Significantly differentially abundant transcripts associated with redox associated proteins (MapMan bin 21) are presented as a heatmap showing relative trans cript abundance according to the green -red scale indicated. Accession numbers of individual transcripts are indicated to the right of are indicated to the right the figure.
